# Metastatic colorectal cancer prior to expanded *RAS* assessment: evidence from long-term outcome analysis of a real-life cohort within a dedicated colorectal cancer unit

**DOI:** 10.1186/s12957-020-01844-5

**Published:** 2020-04-02

**Authors:** Luca Bertero, Rosella Spadi, Simona Osella-Abate, Sara Mariani, Isabella Castellano, Alessandro Gambella, Patrizia Racca, Mario Morino, Paola Cassoni

**Affiliations:** 1 Pathology Unit, Department of Medical Sciences, University of Turin, Turin, Italy; 2 Colorectal Cancer Unit, Città della Salute e della Scienza University Hospital of Turin, Turin, Italy; 3 General Surgery Unit, Department of Surgical Sciences, University of Turin, Turin, Italy

**Keywords:** Colorectal cancer, Metastasis, outcome, KRAS, Cancer unit

## Abstract

**Background:**

Molecular assessment and treatment of metastatic colorectal cancer (mCRC) quickly evolved during the last decades, hampering longitudinal evaluation of prognostic markers. The aim of this study was to evaluate prognostic predictors of long-term survival in a retrospective series of mCRC, treated prior to the expanded *RAS* assessment era.

**Methods:**

mCRC cases treated at the Città della Salute e della Scienza University Hospital (Turin, Italy) between January 2004 and December 2012 were evaluated, including cases with ≥ 5-year follow-up only. Long-term survival was defined as an overall survival (OS) ≥ 4 years based on the observed OS interquartile range values. Univariate/multivariate Cox proportional hazards regression models were performed to assess the prognostic significance of the clinical/biological features, while binary logistic regression models were used to verify their associations with long-term survival.

**Results:**

Two hundred and forty-eight mCRC cases were included and analyzed. Sixty out of two hundred and forty-eight (24%) patients were long-term survivors. Univariate binary logistic regression analysis demonstrated a significant association between long-term survival and age at diagnosis < 65 (OR = 2.28, *p* = 0.007), single metastatic site (OR = 1.89, *p* = 0.039), surgical resection of metastases (OR = 5.30, *p* < 0.001), local non-surgical treatment of metastases (OR = 4.74, *p* < 0.001), and a bevacizumab-including first-line treatment schedule (OR = 2.19, *p* = 0.024). Multivariate binary logistic regression analysis confirmed the prognostic significance of surgical resection of metastases (OR = 3.96, *p* < 0.001), local non-surgical treatment of metastases (OR = 3.32, *p* = 0.001), and of bevacizumab-including first-line treatment schedule (OR = 2.49, *p* = 0.024).

**Conclusion:**

Long-term survival could be achieved in a significant rate of patients with mCRC even in an era of limited molecular characterization. Local treatment of metastases proved to be a significant predictor of long-term survival.

## Background

Colorectal cancer (CRC) is a worldwide leading cause of cancer and cancer-related deaths [1]. Over the past decade, CRC incidence and mortality decreased due to multiple factors: screening protocols implementation, improved surgical techniques, and availability of novel systemic therapeutic options [2]. Nevertheless, metastatic disease is already present at presentation in about 20% of patients (stage IV), while about 30–40% of patients who receive surgical resection will ultimately experience disease recurrence requiring systemic treatments [3].

Although initial disease stage remains the main outcome predictor in CRC, in the precision medicine era, tumor molecular profiling has become of paramount importance [4]. The clinical and histopathological heterogeneity of CRC has been further supported by molecular profiling and specific mutational profiles resulted to be strongly correlated with clinical outcome and response to treatment [5]. Therefore, assessment of specific tumor characteristics and biomarkers [e.g., tumor site (right versus left colon), mismatch repair capability, *KRAS*, *NRAS,* and *BRAF* proto-oncogenes mutational statuses] is now deemed mandatory for treatment selection in patients with mCRC, and the main therapeutic options in this setting include chemotherapy drugs, targeted therapies against the EGFR (Epidermal Growth Factor Receptor), and VEGF (Vascular Endothelial Growth Factor) pathways and immunotherapy [6].

*KRAS* status was the first molecular predictive marker to be routinely assessed in mCRC since several studies proved that *KRAS*-mutant tumors do not benefit from treatment with EGFR inhibitors. Initially, the effect was thought to be restricted to patients with *KRAS* (exon 2) wild-type tumors, but extended *RAS* analyses demonstrated a lack of response also in patients with tumors harboring other *KRAS* (exons 3 and 4) or *NRAS* mutations [7–9]. Expanded *RAS* testing increased the *RAS* mutation rate from 40% to about 55% [10], thus avoiding a potentially ineffective or even harmful anti-EGFR therapy in a significant number of patients. At the same time, extended molecular testing identified the significance of other markers, such as the adverse prognostic effect of *BRAF* mutations at codon 600 [11].

In *RAS*-mutant metastatic CRC (mCRC) patients, first-line therapies usually consist of combined approaches like FOLFOX/CAPOX (5-fluorouracil plus oxaliplatin/capecitabine plus oxaliplatin) or FOLFIRI/CAPIRI (5-fluorouracil plus irinotecan/capecitabine plus irinotecan) plus an additional anti-angiogenetic biologic drug (bevacizumab, ramucirumab, ziv-aflibercept) [12, 13]. In *BRAF*-mutated tumors, a triple chemotherapeutic regimen is usually taken into consideration (FOLFOXIRI: 5-fluorouracil plus oxaliplatin and irinotecan). Conversely, in *RAS* wild-type mCRC, EGFR inhibitors, like cetuximab or panitumumab, are added to conventional chemotherapy drugs [7]. Tumor side also affects the choice of treatment, since left-sided neoplasms shown higher response rates to anti-EGFR treatments [14]. Most recently, novel options became available: regorafenib, a multi-kinase inhibitor, showed efficacy in previously treated mCRC [15–17], whereas programmed cell death protein 1 (PD-1) targeting by immunotherapy (e.g., nivolumab and pembrolizumab) proved to be effective in mCRC with high microsatellite instability (MSI-high) [18, 19].

Despite these efforts, the 5-year survival rate of mCRC remains largely unsatisfying, ranging around just 15% of patients [20]. An improved characterization of long-term survivors is thus warranted to optimize these patients' care, but the quickly evolving landscape hampers comparisons across long time periods.

The aim of this study was therefore to evaluate the long-term survivors’ characteristics in a retrospective series of mCRC treated prior to the expanded *RAS* assessment era and with extended follow up available, to define prognostic markers affecting progression free survival (PFS) and overall survival (OS).

## Methods

### Case series

This is a retrospective observational study on the mCRC cases treated at the Colorectal Cancer Unit of the Città della Salute e della Scienza University Hospital (Turin, Italy) between January 2004 and December 2012. Our study cohort included 248 mCRC (either at the initial diagnosis of CRC or diagnosed with metastatic disease during follow up) patients (≥ 18 year old) who underwent at least one cycle of systemic anti-neoplastic treatment (cytotoxic drug therapy with or without molecular targeted agents). Cases with a follow-up time < 5 years were excluded [median follow up was 7.58 years, IQR (interquartile range) 5.41–9.16 years]. Considered the “real-life”, observational nature of the study no other exclusion criteria were established. The start of the study period was chosen to collect a broadly homogenous cohort, accounting for the introduction of anti-angiogenetic and anti-EGFR agents. Conversely, the accrual was stopped in 2012 prior to the deployment of expanded *RAS* assessment protocols and to achieve a sufficient follow-up. To define long-term survivors (LTS), a survival time ≥ 4 years was selected based upon the observed overall survival IQR values (1.54–4.00 years): this cutoff value enabled to have a representative number of cases (about one quarter of the whole series) available for the subsequent analyses while also being a clinically meaningful time period.

Clinical information was gathered from patients’ charts, hospital discharge forms, imaging repositories, and contacts with general practitioners. Collected data included gender, age at diagnosis, tumor side, *KRAS* status (exon 2, codons 12, and 13), number and sites of metastases, details regarding systemic and locoregional (surgery and/or radiofrequency) treatments, and disease outcome (PFS and OS). Chemotherapy schedules comprised a combination of drugs including fluorouracil, capecitabine, oxaliplatin, irinotecan, mitomycin, bevacizumab, cetuximab, and panitumumab according to the disease setting and available clinical trials.

The study was conducted in accordance with The Code of Ethics of the World Medical Association (Declaration of Helsinki) for experiments involving humans and within the guidelines and regulations defined by the Research Ethics Committee of the University of Turin. This study was approved by the Research Ethics Committee of the University of Turin; considered the retrospective nature of the research protocol and that it had no impact at all on patients’ care, no specific written informed consent was required.

### Statistical analyses

All analyses were performed using the Stata/MP 15.0 Statistical Software (StataCorp, College Station, TX, USA). Continuous variables were summarized by mean and standard deviation (SD); whereas for categorical variables, the frequency and percentage were provided. The characteristics at diagnosis were compared using the chi-square test for categorical variables and the *T* test or ANOVA test for continuous ones.

The follow-up time, calculated with the reverse Kaplan–Meier method, was summarized as median and interquartile range (IQR). The survival times were measured from the start of treatment at metastasis diagnosis until disease progression (PFS) and death from any cause (OS); patients lost at follow-up (after a minimum of 5 years as per inclusion criteria) were censored on the last follow-up date. Survival curves were estimated with the Kaplan–Meier method and compared by log-rank test. The impact of possible confounders was explored by univariate and multivariate Cox proportional hazards regression models, including clinical/biological features as covariates. The proportional hazards assumption was tested for all the endpoints. Hazard ratios (HR) and 95% confidence intervals (95% CI) were estimated. Univariate/multivariate binary logistic regression models were performed using long-term survival as the dependent variable (yes or no) and patient/tumor characteristics as covariates. Odds ratios and 95% CIs were estimated. The Hosmer–Lemeshow goodness-of-fit test was used to determine whether the model adequately described the data. Differences were considered significant when *p* < 0.05 for reported two-sided *p* values.

## Results

### Patients’ clinical and pathological characteristics

The clinical and pathological characteristics of patients are presented in Table 1. The median age was 65 years (range 32–82), and the male to female ratio was 1.32. Primary tumor was right-sided in 83/248 (33%) cases, left-sided in 97/248 (39%), and localized in the rectum in 68/248 (28%). A *KRAS* mutation was detected in 45.2% of patients (based on codon 2 assessment only, i.e., before expanded *RAS* protocols).

**Table 1:** Clinico-pathological characteristics according to long-term survival status

		Total (*n* = 248)	Survivors < 4 years (*n* = 188)	Long-term survivors (≥ 4 years) (*n* = 60)	*p* value
**Gender**	**Female**	102	79	23	0.613
	**Male**	146	109	37	
**Age at diagnosis**	**Median (range) (years)**	65 (32–82)	66 (37–82)	60 (32-82)	**0.012**
**Age at metastasis**	**Median (range) (years)**	65 (35–83)	66 (37–83)	62 (35–83)	**0.026**
**Mutational status*KRAS*(exon 2)**	**WT**	136	100	36	0.651
	**Codon 12 mutation**	88	69	19	
	**Codon 13 mutation**	24	19	5	
**Grading (not available 5; surgical resection of primary tumor not performed 30)**	**1**	7	5	2	0.646
	**2**	140	102	38	
	**3**	66	52	14	
**pT** **(not available 1; surgical resection of primary tumor not performed 30)**	**1**	3	1	2	0.052
	**2**	11	7	4	
	**3**	136	97	39	
	**4**	72	55	12	
**Primary site**	**Right side**	83	69	14	0.150
	**Left side**	97	71	26	
	**Rectum**	68	48	20	
**Primary site**	**Right side**	83	69	14	0.056
	**Left side + Rectum**	165	119	46	
**Metastasis at diagnosis**	**No**	89	63	26	0.167
	**Yes**	159	125	34	
**Metastatic site**	**Liver**	98	70	28	0.301
	**Lung**	18	12	6	
	**Peritoneum**	15	11	4	
	**Lymph nodes**	5	3	2	
	**Multiple sites**	111	91	20	
**Number of metastatic sites**	**Single site**	136	96	40	**0.038**
	**Multiple sites**	111	91	20	
**Surgical resection of primary tumor**	**No**	30	27	3	0.053
	**Yes**	218	161	57	
**Adjuvant treatment**	**No**	169	132	37	0.216
	**Yes**	79	56	23	
**Metastasis surgical resection**	**No**	159	140	22	**<0.001**
	**Yes**	89	48	38	
**Local non-surgical treatment of metastasis**	**No**	174	148	26	**<0.001**
	**Yes**	74	40	34	

The overall number of treatment lines are reported in Table 2. A higher rate of patients with an OS < 4 years received only one line of treatment (18% versus 8%); conversely, more long-term survivors (≥ 4 years) received 4 lines (47% versus 27%).

**Table 2:** Lines of treatment

		Total (*n* = 248)	Survivors < 4 years (*n* = 188)	Long-term survivors (≥ 4 years) (*n* = 60)
**Number of lines of treatment**	**1**	39 (16%)	34 (18%)	5 (8%)
	**2**	63 (25%)	51 (27%)	12 (20%)
	**3**	67 (27%)	52 (28%)	15 (25%)
	**4**	79 (32%)	51 (27%)	28 (47%)

### Overall outcome analysis

**Fig. 1:**
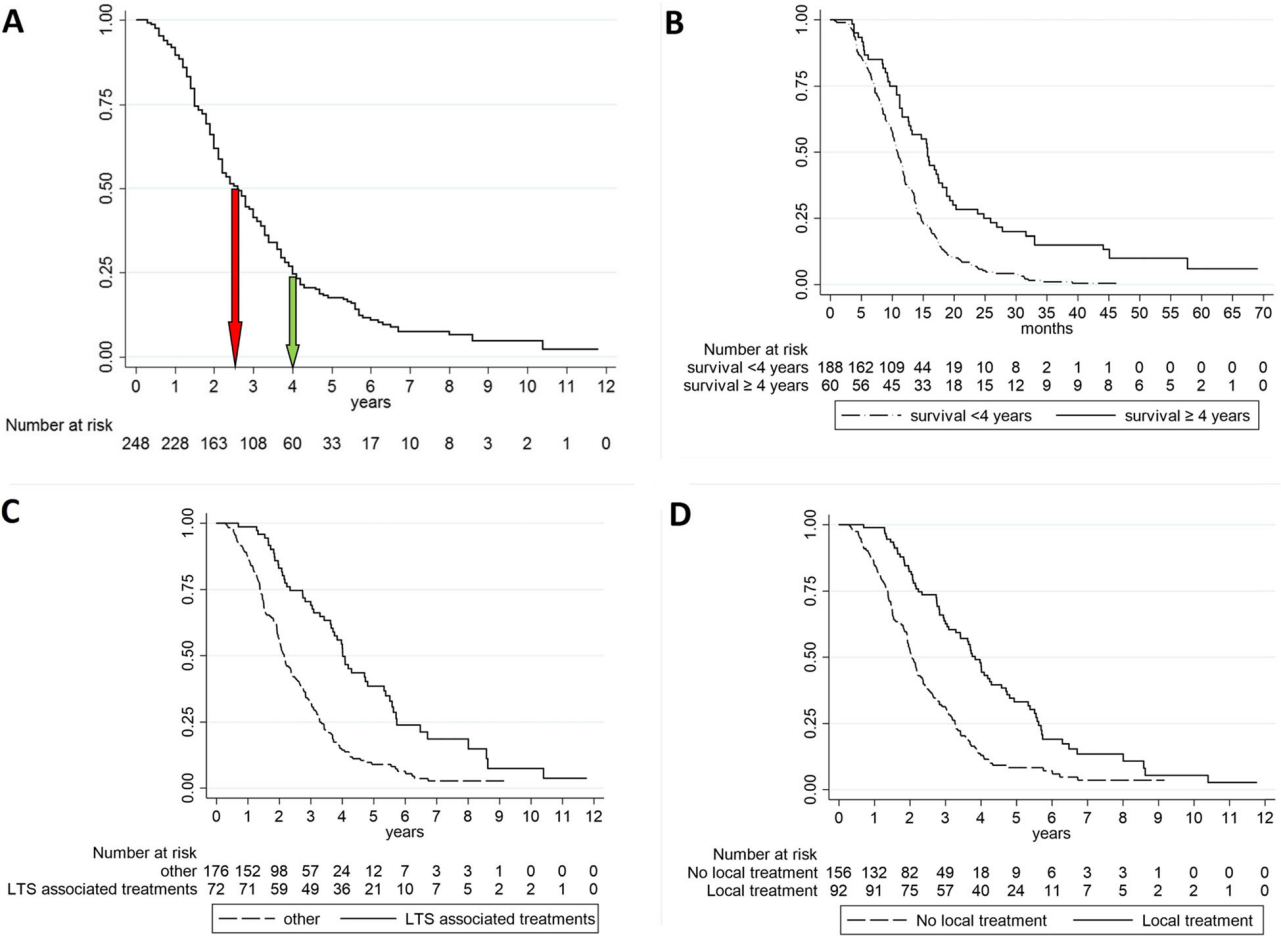
**a** Kaplan–Meier curve of OS. Median OS time was 2.62 years (red arrow), but survival was longer than 4 years in 24% of patients (green arrow). **b** Kaplan–Meier curve of 1st-line treatment PFS according to LTS. **c** Kaplan–Meier curve of OS according to LTS-associated treatments (bevacizumab-including 1st-line regimen, surgical resection of metastases, local non-surgical treatment of metastases). **d** Kaplan–Meier curve of OS according to local treatment of metastases (either by surgery or other approaches)

Median overall PFS after first-line treatment was 11.6 months (IQR 7.5–16.7 months), while median OS time was 2.62 years (IQR 1.54–4.00 years), and 60 out of 248 (24%) patients were long-term survivors (OS ≥ 4 years) (Fig. 1a).

At univariate analysis (Table 3), the variables significantly associated with first-line PFS were age at diagnosis, age at metastasis, surgical resection of primary tumor, surgical resection of metastasis, local non-surgical treatment of metastasis, and bevacizumab administration at first line. Conversely, no correlation was found between gender, tumor site (right sided versus other), site of metastases, *KRAS* mutational status (based on exon 2 evaluation), or adjuvant treatment.

**Table 3:** PFS and OS univariate Cox regression analyses of the whole series

		First-line PFS			OS		
Variable		HR	95% CI	p	HR	95% CI	*p*
**Gender**		1.00	0.77–1.29	0.991	1.11	0.84–1.46	0.438
**Age at diagnosis**		1.02	1.01–1.03	**0.001**	1.04	1.02–1.05	**< 0.001**
**Age at metastasis**		1.02	1.00–1.03	**0.003**	1.04	1.02–1.05	**< 0.001**
**Age at metastasis > 60 years**		1.40	1.07–1.65	**0.015**	1.76	1.30–2.38	**< 0.001**
**Tumor grade**	**1**	1			1		
	**2**	1.24	0.58–2.66	0.570	0.67	0.31–1.45	0.321
	**3**	1.37	0.63–3.00	0.426	1.04	0.47–2.28	0.921
**pT**	**1**	1			1		
	**2**	0.82	0.18–3.74	0.806	1.27	0.27–6.00	0.764
	**3**	1.04	0.25–4.21	0.956	1.92	0.47–7.81	0.360
	**4**	1.31	0.32–5.36	0.706	2.76	0.67–11.39	0.158
**Right sided tumor versus other (left sided and rectum)**		1.24	0.94–1.61	0.115	1.36	1.03–1.80	**0.030**
**Metastasis site**	**Liver**	1			1		
	**Other sites**	1.11	0.76–1.64	0.579	1.05	0.71–1.57	0.796
	**Multiple sites**	1.29	0.97–1.70	0.070	1.17	0.88–1.57	0.277
**Multiple site versus single site metastases**		1.25	0.91.62	0.082	1.16	0.89–1.52	0.274
***KRAS*mutant (exon 2) versus wild-type**		1.25	0.97–1.61	0.083	1.32	1.01–1.72	**0.039**
**First-line therapy**	**Chemotherapy plus bevacizumab**	1			1		
	**Chemotherapy plus anti-EGFR**	1.03	0.49–2.13	0.933	1.05	0.45–2.41	0.909
	**Chemotherapy alone**	1.44	1.09–1.88	**0.008**	0.91	0.68–1.22	0.544
**First-line therapy**	**Chemotherapy plus bevacizumab versus other**	0.71	0.54–0.93	**0.012**	1.09	0.81–1.45	0.568
**Surgical resection of primary tumor (yes versus no)**		0.59	0.40-0.88	**0.009**	0.64	0.42–0.96	**0.030**
**Surgical resection of metastasis (yes versus no)**		0.55	0.41–0.72	**< 0.001**	0.44	0.32–0.59	**< 0.001**
**Local non-surgical treatment of metastasis (yes versus no)**		0.60	0.45–0.80	**< 0.001**	0.47	0.35–0.64	**< 0.001**

Variables affecting OS were age at diagnosis, age at metastasis, right-sided tumor, *KRAS* exon 2 mutational status, surgical resection of primary tumor, surgical resection of metastasis, and local non-surgical treatment of metastasis. No correlation was found with gender, site of metastases, adjuvant treatment, or a bevacizumab-including first-line regimen.

Multivariable Cox regression analysis was stratified by local non-surgical treatment of metastases because this covariate violated the proportional hazards assumption. Surgical resection of metastasis was confirmed to be a significant predictor in terms of first line PFS and/or OS by multivariate analyses (Table 4), as well as first-line treatment including bevacizumab, age at metastasis diagnosis, and right sided tumor.

**Table 4:** PFS and OS multivariate Cox regression analyses of the whole series

	First line PFS			OS		
Variable	HR	95% CI	3.3. *p*	HR	95% CI	*p*
**Gender**	0.86	0.69–1.08	0.199	1.21	0.96–1.54	0.109
**Age at metastasis**	1.01	0.99–1.02	0.180	1.02	1.00–1.04	**0.009**
**Chemotherapy plus bevacizumab versus other**	0.70	0.66–0.74	**< 0.001**	–	–	–
**Surgical resection of metastasis (Yes versus No)**	0.57	0.44–0.71	**< 0.001**	0.49	0.39–0.61	**< 0.001**
**Right-sided tumor versus other (left-sided and rectum)**	–	–	–	1.18	1.00–1.40	**0.048**
***KRAS*mutant (exon 2) versus wild-type**	–	–	–	1.28	0.75–2.18	0.365

Standard error adjusted for 2 clusters in local treatment

### Characteristics of long-term survivors

No significant differences were observed in terms of gender, primary tumor site, histological grade, *KRAS* mutational status, or adjuvant treatment according to long-term survival status (Table 1). Conversely, long-term survivors (≥ 4 years) showed a lower median age at diagnosis (60 versus 66, *p* = 0.012) and at metastasis diagnosis (62 versus 66 years, *p* = 0.026), a lower rate of multiple metastatic sites (33.3 % versus 48.4%, *p* = 0.038), and higher rates of metastasis surgical treatment (63% versus 26%, *p* < 0.001) and/or local non-surgical metastasis treatment (57% versus 21%, *p* < 0.001).

Long-term survivors also showed a significant longer first-line treatment median PFS compared with other patients (15.7 versus 10.8 months, respectively, *p* = 0.0001) (Table 5 and Fig. 1b). This statistical difference was maintained in subsequent treatment lines as well (Table 5).

**Table 5:** PFS according to lines of treatment

		Survivors < 4 years (*n* = 188)		Long-term survivors (≥ 4 years) (*n* = 60)		*p* value
		*n*	PFS (months) (IQR)	*n*	PFS (months) (IQR)	
**1st-line overall**	**Median (interval) (months)**	188	10.8 (0.7–46.6)	60	15.7 (3.5–69)	**0.0001**
**Type of treatment**	**Chemotherapy plus bevacizumab**	71	12 (1–46.6)	13	25.9 (11.6–69)	
	**Chemotherapy plus anti-EGFR**	7	16.1 (6.5–32)	1	5.1	
	**Chemotherapy alone**	110	10.1 (0.7–39.1)	46	13.1 (3.5–60.8)	
**2nd-line overall**	**Median (interval) (months)**	154	4.4 (0.2–21.9)	51	9.8 (2.0–47.8)	**< 0.001**
**Type of treatment**	**Chemotherapy plus bevacizumab**	25	5.7 (1.3–19.4)	8	9.45 (2.1–24.3)	
	**Chemotherapy plus anti-EGFR**	28	3.05 (0.2–14.1)	5	10.3 (2.5–24.7)	
	**Chemotherapy alone**	101	4.3 (0.2–21.9)	38	9.65 (2–47.8)	
**3rd-line overall**	**Median (interval) (months)**	103	3.2 (0.6–20.2)	43	7.6 (0.2–33)	**< 0.001**
**Type of treatment**	**Chemotherapy plus bevacizumab**	1	4.65 (2.6–6.7)	3	8 (7.3–16.2)	
	**Chemotherapy plus anti-EGFR**	33	3.45 (0.6–9.9)	17	8.1 (1.9–33)	
	**Chemotherapy alone**	69	3.1 (0.4–20.2)	23	7.1 (0.2–28)	
**4th-line overall**	**Median (interval) (months)**	51	2.6 (0.6–8.8)	28	7.7 (2.2–17.9)	**< 0.001**
**Type of treatment**	**Chemotherapy plus bevacizumab**	0	–	1	12.1	
	**Chemotherapy plus anti-EGFR**	5	4.1 (2.6–8.8)	6	15.5 (8.2–17.9)	
	**Chemotherapy alone**	46	2.6 (0.6–7)	21	6.35 (2.2–17.1)	

### Outcome analysis of long-term survivors

Based on the previous results in terms of variables affecting outcome, univariate binary logistic regression analysis was performed (Table 6): a significant association was observed between long-term survival and age at diagnosis < 65, a single metastatic site, surgical resection of metastases, local non-surgical treatment of metastases, and a bevacizumab-including first-line treatment schedule.

**Table 6:** Univariate binary logistic regression analysis of characteristics associated with long-term survival

		Long-term survival		
Variable		OR	95% CI	*p*
**Gender**		1.16	0.64–2.11	0.613
**Age at metastasis ≤ 65 years (yes versus no)**		2.28	1.25–4.15	**0.007**
**Right-sided tumor versus other (left-sided and rectum)**		0.52	0.26–1.02	0.058
**Single site metastases versus multiple sites**		1.89	1.03–3.48	**0.039**
***KRAS*mutant (exon 2) versus wild-type**		0.74	0.41–1.33	0.321
**First-line therapy**	**Chemotherapy plus bevacizumab versus other**	2.19	1.11–4.33	**0.024**
**Surgical resection of primary tumor (yes versus no)**		3.18	0.93–10.9	0.065
**Surgical resection of metastasis (yes versus no)**		5.30	2.80–10	**< 0.001**
**Local non-surgical treatment of metastasis (yes versus no)**		4.74	0.11–0.27	**< 0.001**
**Adjuvant treatment (yes versus no)**		1.46	0.80–2.69	0.217

Multivariate binary logistic regression analysis (Table 7) confirmed the significant association between long-term survival and surgical resection of metastases, local non-surgical treatment of metastases, and a bevacizumab-including first-line treatment schedule.

**Table 7:** Multivariate binary logistic regression analysis of characteristics associated with long-term survival

		Long-term survival		
Variable		OR	95% CI	*p*
**Age at metastasis ≤ 65 years (yes versus no)**		1.95	0.96–3.97	0.064
**Single site metastases versus multiple sites**		1.76	0.87–3.58	0.114
**First-line therapy**	**Chemotherapy plus bevacizumab versus other**	2.49	1.13–5.52	**0.024**
**Surgical resection of metastasis (yes versus no)**		3.96	1.96–8.02	**< 0.001**
**Local non-surgical treatment of metastasis (yes versus no)**		3.32	1.64–6.71	**0.001**

Based upon these results, we wanted to verify the association between these long-term survival (LTS) associated treatments and OS in the whole series. Considered that both surgical and non-surgical local treatment of metastases were aimed at the local control of a systemic disease, we considered these two treatments as equivalent. Thus, the identified LTS-associated treatments were (i) bevacizumab at first line and (ii) surgical resection of metastasis and/or local non-surgical treatment of metastasis. Kaplan–Meier analysis of the whole series (*n* = 248) showed a significantly longer OS in patients treated with LTS-associated treatments (log-rank test *p* < 0.001): 3.92 years median (IQR 0.69–11.7) versus 2.15 years median (IQR 0.30–9.17) (Fig. 1c). Considering local treatment of metastases exclusively (irrespectively if by surgery or other approaches), an association with longer OS was still confirmed (log-rank test < 0.001): 3.83 years median (IQR 2.23–5.26) versus 2.05 years median (IQR 1.37–3.26) (Fig. 1d).

Finally, considered the prognostic role of local metastasis treatment, we verified the characteristics of metastatic disease in the series (Table 8). Overall, a higher rate of single metastases was found within long-term survivors compared with other patients (67% versus 52%). Among patients with single site metastases, a higher rate of long-term survivors received local treatments (75% versus 22% in patients with < 4 years survival).

**Table 8:** Metastatic sites according to survival status and local treatment of metastases

	Survivors < 4 years (*n* = 188)			Long-term survivors (≥ 4 years) (*n* = 60)		
Number of metastatic sites	Local treatments for metastases (*n* = 50)	No local treatments (*n* = 138)	Total	Local treatments for metastases (*n* = 42)	No local treatments (*n* = 18)	Total
**Single site**	**21**	**76**	**97**	**30**	**10**	**40**
**Multiple sites**	**29**	**62**	**91**	**12**	**8**	**20**

## Discussion

In the present study, outcome analysis of a real-life series of mCRC treated prior to expanded *KRAS* assessment and with long-term follow-up was performed: a high rate (24%) of long-surviving patients (≥ 4 years) was observed, and their characteristics were evaluated. Local treatment of metastases (surgical or non-surgical) and a first-line treatment including bevacizumab resulted significantly associated with PFS/OS and long-term survival at univariate and multivariate analyses.

Overall characteristics of the series were as expected: tumor involved right colon in 1/3 of cases, while the neoplasm was left-sided (left colon or rectum) in the remaining 2/3 [21]. Molecular status (*KRAS* mutation rate of about 45%) was consistent with the results observed prior to expanded *KRAS* evaluation [10]. Most of the patients had synchronous metastases at diagnosis (64%), and the majority of them had single site involvement (55%).

mCRC is a leading cause of cancer-related death despite our fast evolving knowledge regarding the molecular inner-workings of this disease and the availability of novel therapeutic options. Thanks to the introduction of the FOLFOX/FOLFIRI schedules, median OS reached the 2 years milestone, while addition of anti-angiogenetic and anti-EGFR biological therapies led to a further improvement to about 30 months [22]. Despite the real-life retrospective nature of our series and the time period analyzed, we observed a median OS of more than 30 months which is in line or even superior to what could have been expected, considered the analyzed years and the now outdated molecular profiling based on *KRAS* exon 2 evaluation only. This is a first interesting result and a possible explanation of this good overall outcome could be the specific study setting: a tertiary university hospital with a dedicated colorectal cancer unit. Management of patients with colorectal cancer by multidisciplinary teams proved to be especially relevant for advanced disease [23] and was found to be associated with improved outcomes in multiple reports [24–28].

Lower age at metastasis (*p* = 0.009), surgical resection of metastases (*p* < 0.001), and a left-sided tumor (*p* = 0.048) were associated with longer OS by multivariate Cox regression analysis stratified by local treatment. Data accrued during the last few years showed an association between right sided primary tumors and poorer outcome even when accounting for the specific tumor molecular profile (like *RAS* status) or other variables [14, 29, 30]. This finding could be potentially due to the different embryologic origin of the right versus left colon and is now being accounted for when designing new clinical trials. Our results confirm these findings in a large series with extended follow-up.

Focusing on long-term survivors (≥ 4 years), this group differed in terms of (i) lower age at initial diagnosis (60 versus 66, *p* = 0.012) and at metastasis (62 versus 66 years, *p* = 0.026); (ii) lower rate of multiple metastatic sites (33.3 % versus 48.4%, *p* = 0.038); (iii) higher rates of surgical (63% versus 26%, *p* < 0.001) and/or local non-surgical metastasis treatment (57% versus 21%, *p* < 0.001); (iv) a significantly longer first-line treatment median PFS (15.7 versus 10.8 months respectively, *p* = 0.0001). Although this latter association could be expected and considered an example of responder bias, it is interesting to note that the statistically significant difference was confirmed in all subsequent treatment lines as well; thus, the sequential PFSs observed during the disease course seem to remain informative to estimate patients’ outcome.

Regarding outcome analysis, the variables which showed the strongest association with long-term survival were the local treatments of metastases (either surgically or not). This finding was confirmed by multivariate binary regression logistic analysis. The availability of a specific dedicated multidisciplinary colorectal team probably had a role in enabling this kind of treatment in such a significant group of patients (37%, 92/248), including 37% (41/111) of those with multiple sites involvement.

Our results encourage a focused, active treatment of metastases if clinically feasible, even when multiple sites involvement is present. Indeed, it has been long known that surgical resection of liver metastasis in mCRC can achieve long-term survival or cure in a high rate of patients (even > 50%), especially in oligometastatic patients with liver involvement only, but the present results support that a long-term benefit can be achieved also in case of metastatic spread to multiple sites [31, 32]. This observation is consistent with the recent trial results in patients with unresectable metastases which demonstrated a significantly longer OS thanks to a combined approach (systemic treatment plus aggressive local treatment by radiofrequency ablation ± resection) compared with systemic therapy alone [33]. Conversely, randomized trials comparing surgical and non-surgical approaches are lacking. In our cohort, both approaches were associated with an improved survival, but no conclusions can be inferred regarding the superiority of a specific option since many patients received both the treatments.

Bevacizumab at first line was the second variable which resulted associated with long-term survival and first line PFS despite a non-significant finding in terms of OS. This benefit is consistent with literature data, and the missing association with OS could be due to the limited sample size of this group of patients [34]. These results highlight the importance of long-term outcome analysis to comprehensively assess the efficacy of different prognostic factors and treatments, both in terms of their combinations and sequencing. The potential synergistic effect of a bevacizumab-including regimen followed by metastasis surgical resection should also be kept in mind considered the recent data suggesting the positive impact of this approach on OS of mCRC [35, 36].

Nevertheless, these findings must be interpreted cautiously due to the retrospective, real-life nature of the study and its consequent, intrinsic limitations. During the study period, some variations occurred both in terms of the routine treatment protocols and the available clinical trials. The limited sample size, in particular of the long-survivors group, is a second limitation. Although this drawback could have been tackled by broadening the time period selected to collect the cases, it would have significantly increased the heterogeneity in terms of molecular assessment, treatments, and patients’ management; thus, we tried to reach a compromise between numerosity and heterogeneity to enable a meaningful longitudinal analysis. Nevertheless, the characteristics of such a real-life retrospective cohort allowed to identify variables which were significantly associated with outcome despite these changes over time [37]. Another relevant perspective, which would have been interested to investigate, is quality of life and patient-reported outcomes measures which are especially relevant in long-term cancer survivors [38]. This shortcoming could be addressed by implementing a brief assessment of these parameters into the routine clinical evaluation, but these efforts are hindered by the ever-increasing demands on healthcare staff.

Further studies, based on more recent cohorts, will allow to verify the significance of the prognostic markers here identified within specific mCRC molecular subgroups and their correlations with the newly introduced drugs and regimens. As shown during the last few years in melanoma or lung adenocarcinoma, new treatments like immunotherapy and/or targeted therapies can dramatically reshape the therapeutic approaches, even in patients with advanced/metastatic disease [39, 40]. This is another promising perspective of tailored medicine which shall not be undervalued: precision oncology should not only rest on new molecularly driven drugs, but also on the optimization of the already validated treatments within the different clinical settings and for each single patient.

## Conclusions

Our study shows that long-term survival could often be achieved in mCRC even in an era of limited molecular characterization. Although the clinical setting of the present study (a tertiary-level university hospital) has likely played a role in achieving these overall results, local treatment of metastases proved to be a clear predictor of long-term survival. These data will also help compare and interpret the long-term results of the new cohorts of patients which are now being treated according to more extensive molecular profiling and novel protocols.

## Data Availability

The datasets generated and/or analyzed during the current study are not publicly available to protect patients’ privacy, but are available from the corresponding author upon reasonable request.

## Abbreviations

mCRC: Metastatic colorectal cancer
CRC: Colorectal cancer
EGFR: Epidermal Growth Factor Receptor
VEGF: Vascular Endothelial Growth Factor
FOLFOX/CAPOX: 5-fluorouracil plus oxaliplatin/capecitabine plus oxaliplatin
FOLFIRI/CAPIRI: 5-fluorouracil plus irinotecan/capecitabine plus irinotecan
FOLFOXIRI: 5-fluorouracil plus oxaliplatin and irinotecan
MSI-high: High microsatellite instability
PFS: Progression free survival
OS: Overall survival
SD: Standard deviation
IQR: Interquartile range
HR: Hazard ratios
95% CI: 95% confidence intervals
LTS: Long-term survival
